# Cardiovascular and cerebral hemodynamics during exercise and recovery in obese individuals as a function of their fitness status

**DOI:** 10.14814/phy2.13321

**Published:** 2017-06-22

**Authors:** Mathieu Gayda, Gabriel Lapierre, Olivier Dupuy, Sarah Fraser, Louis Bherer, Martin Juneau, Vincent Gremeaux, Anil Nigam

**Affiliations:** ^1^ Cardiovascular Prevention and Rehabilitation Centre (ÉPIC)Montreal Heart Institute and Université de MontréalMontrealQuebecCanada; ^2^ Research CenterMontreal Heart Institute and Université de MontréalMontrealQuebecCanada; ^3^ Department of MedicineFaculty of MedicineUniversité de MontréalMontrealQuebecCanada; ^4^ Department of KinesiologyUniversité de MontréalMontrealQuebecCanada; ^5^ LaboratoryMOVE (EA6314)Faculty of Sport SciencesUniversity of PoitiersPoitiersFrance; ^6^ Interdisciplinary School of Health SciencesUniversity of OttawaOttawaOntarioCanada; ^7^ Plateforme d'investigation technologiqueINSERM CIC 1432CHU DijonDijonFrance

**Keywords:** Cardiac and cerebral hemodynamics, exercise, obesity, recovery

## Abstract

The aim of this study was to compare cardiovascular hemodynamics and cerebral oxygenation/perfusion (COP) during and after maximal incremental exercise in obese individuals according to their aerobic fitness versus age‐matched healthy controls (AMHC). Fifty‐four middle–aged obese (OB) and 16 AMHC were recruited. Maximal cardiopulmonary function (gas exchange analysis), cardiac hemodynamics (impedance cardiography), and left frontal COP (near‐infrared spectroscopy: NIRS) were measured continuously during a maximal incremental ergocycle test. During recovery, reoxygenation/perfusion rate (ROPR: oxyhemoglobin: ΔO_2_Hb, deoxyhemoglobin: ΔHHb and total hemoglobin: ΔtHb; with NIRS) was also measured. Obese participants (OB,* n* = 54) were divided into two groups according to the median $\dot{V}O_{2}$ peak: the low‐fit obese (LF‐OB,* n* = 27) and the high‐fit obese (HF‐OB,* n* = 27). During exercise, end tidal pressure of CO_2_ (PETCO_2_), and COP (ΔO_2_Hb, ΔHHb and ΔtHb) did not differ between groups (OB, LF‐OB, HF‐OB, AMHC). During recovery, PETCO_2_ and ROPR (ΔO_2_Hb, ΔHHb and ΔtHb) were similar between the groups (OB, LF‐OB, HF‐OB, AMHC). During exercise and recovery, cardiac index was lower (*P* < 0.05) in LF‐OB versus the other two groups (HF‐OB, AMHC). As well, systolic blood pressure was higher during exercise in the OB, LF‐OB and HF‐OB groups versus AMHC (*P* < 0.05). When compared to AMHC, obese individuals (OB, LF‐OB, HF‐OB) have a similar cerebral vasoreactivity by CO_2_ and cerebral hemodynamics during exercise and recovery, but a higher systolic blood pressure during exercise. Higher fitness in obese subjects (HF‐OB) seems to preserve their cardiopulmonary and cardiac function during exercise and recovery.

## Introduction

The prevalence of obesity in adults has dramatically increased, reaching 25–39.5% in Canada and USA (Nguyen and Lau 2012; Ogden et al. 2013). Obesity is associated with important comorbidities like cardiovascular diseases, hypertension and diabetes, but also with a higher risk of cerebrovascular diseases such as stroke, dementia and/or Alzheimer disease (Wilson et al. 2008; Toda et al. 2014). Obesity is associated with resting cerebral abnormalities that include: increased brain arterial stiffness/pulsatility, reduced cerebral endothelial function, reduced cerebral blood flow and cardiac diastolic and/or systolic dysfunction (Furtner et al. 2009; Toda et al. 2014). Early in obesity (younger adults), there is an adaptive cardiac mechanism, with an increased blood volume, cardiac preload and enlargement and thickening of the left ventricles measured at rest, therefore resting systolic function can be increased and/or preserved (Artham et al. 2008; Vella et al. 2012). With obesity duration (older adults), cardiac adaptations can lead to pathological eccentric remodeling associated with cardiac diastolic and systolic dysfunction (Artham et al. 2008; Vella et al. 2012; Crendal et al. 2013), this could have an impact on cardiovascular function during exercise in older obese adults. Some previous studies (with only one reported in middle‐aged obese adults) demonstrated a reduced normalized maximal aerobic power ($\dot{V}O_{2}$ peak divided by lean body mass) (Vella et al. 2012; Fournier et al. 2014) associated with an impaired cardiac systolic function (Vella et al. 2012; Fournier et al. 2014) and muscle oxygen extraction (C(a‐v)O_2_) (Vella et al. 2011, 2012; Fournier et al. 2014) in obese subjects as compared to age‐matched nonobese healthy controls (AMHC) during incremental exercise. Similarly to the cardiovascular function, very few studies are reported on the impact of obesity on cerebral hemodynamics during exercise and recovery, and none were performed in older obese adults. Conflicting results on cerebral hemodynamics during exercise were recently reported in young obese men versus AMHC (Cavuoto and Maikala 2015, 2016). One study reported a similar cerebral oxygenation‐perfusion (measured by near‐infra red spectroscopy: NIRS) during submaximal cycling exercise in young obese men versus AMHC (Cavuoto and Maikala 2016), while the same authors showed a reduced cerebral oxygenation (measured by NIRS) during repetitive incremental lifting to exhaustion in a similar cohort (Cavuoto and Maikala 2015). These discrepant results might come from confounding variables. In healthy subjects, principal cardiovascular parameters that modulate cerebral oxygenation‐perfusion (COP) during exercise are arterial blood pressure, carbon dioxide (PaCO_2_) and cardiac output (Ogoh and Ainslie 2009). As well, other factors such as cardiovascular disease and aerobic fitness ($\dot{V}O_{2}$peak) can influence COP during exercise. For example, a higher COP has been observed in the higher fit ($\dot{V}O_{2}$peak) younger and older healthy subjects (Rooks et al. 2010; Bailey et al. 2013; Brugniaux et al. 2014). As well, cardiac patients have a lower COP during exercise and recovery compared to healthy controls, in relation to a reduced $\dot{V}O_{2}$peak and cardiac function (Koike et al. 2004a, 2004b; Fu et al. 2013; Gayda et al. 2016). Obesity in older adults can be associated with a reduced $\dot{V}O_{2}$ peak and impaired cardiac systolic function (Vella et al. 2012; Fournier et al. 2014) which could reduce COP during exercise and its recovery, particularly in less fit obese subjects.

A better understanding of the interrelationships between $\dot{V}O_{2}$ peak, COP, cardiovascular hemodynamics and fitness level during and after exercise might provide promising future intervention avenues for individuals with obesity. To the best of our knowledge, no previous study has compared cardiovascular hemodynamics and COP during maximal incremental exercise and recovery in older obese subjects. In addition, the influence of initial aerobic fitness level ($\dot{V}O_{2}$ peak) on cardiovascular hemodynamics and COP during exercise and recovery is unknown in older obese subjects. Therefore, the aim of this study was to compare cardiovascular hemodynamics and COP during and after maximal incremental exercise in obese individuals compared to nonobese aged‐matched healthy controls (AMHC) while taking into account aerobic fitness level ($\dot{V}O_{2}$ peak). We hypothesized that cardiovascular hemodynamics and COP during maximal exercise and recovery will be reduced in the obese subjects, particularly the less fit.

## Methods

### Subjects

A total of 70 adults were enrolled from the Cardiovascular Prevention and Rehabilitation Centre of the Montreal Heart Institute, including 16 AMHC (64 ± 4 years) and 54 obese subjects (OB) (62 ± 6 years). The whole OB group (*n* = 54) was divided into two groups according to their median $\dot{V}O_{2}$ peak: the low‐fit obese group (LF‐OB: *n* = 27) and the high‐fit obese group (HF‐OB: *n* = 27). For AMHC, inclusion criteria were: age >18 years, normal adiposity (<25% for men and <35% for women) (Cornier et al. 2011; Dalzill et al. 2014), no cardiovascular (CV) risk factors and no evidence of cardiovascular disease. For OB, inclusion criteria were: age >18 years, to be obese defined by a body fat mass percentage > 25% for men and >35% for women (Cornier et al. 2011; Dalzill et al. 2014), no evidence of cardiovascular disease. The CV risk factors were defined as follows (Dalzill et al. 2014): diabetes was defined as a prior diagnosis of diabetes along with a fasting glucose >7.1 mmol/L and an HbA_1c_ level > 0.06 or a treatment with an hypoglycemic agent. Hypertension was defined as a prior diagnosis of hypertension with blood pressure >130/85 mm Hg or antihypertensive treatment. Active smoking was defined as smoking ≥1 cigarette, cigar, or pipe per day. Dyslipidemia was defined as total cholesterol ≥6.2 mmol/L, low‐density lipoprotein cholesterol ≥4.2 mmol/L, or a total/high‐density lipoprotein cholesterol ratio ≥4.7 or statin treatment. Exclusion criteria for OB and AMHC were: recent acute coronary syndrome (<3 months), significant resting electrocardiogram (ECG) abnormality, history of ventricular arrhythmias or congestive heart failure, uncontrolled hypertension, recent bypass surgery intervention <3 months, recent percutaneous transluminal coronary angioplasty <6 months, left ventricular ejection fraction <45%, pacemaker or implantable cardioverter defibrillator, recent modification of medication <2 weeks, and musculoskeletal conditions making exercise on ergocycle contraindicated (Dalzill et al. 2014). All subjects underwent a baseline evaluation that included a medical history, a physical examination with measurement of height and weight, body composition (bioimpedance, Tanita, model BC418, Japan) and fasting blood sample (glucose and lipid profile) (Dalzill et al. 2014; Drigny et al. 2014). They performed a maximal cardiopulmonary exercise test (CPET) with continuous measurement of gas exchange, cerebral hemodynamics with NIRS and cardiac hemodynamics (impedance cardiography) (Gayda et al. 2012, 2016; Gremeaux et al. 2012; Drigny et al. 2014). All participants gave their written informed consent and the protocol was approved by the Ethics Committee of Montreal Heart Institute. The study was registered on ClinicalTrials.gov under identifier number: NCT03018561.

### Measurements

#### Maximal cardiopulmonary exercise testing

This test was performed on a ergocycle (Ergoline 800S, Bitz, Germany), with an individualized protocol that included: a 3‐min warm up at 20 Watts, followed by a power increase in 10 to 20 Watts/min until exhaustion at a pedaling speed > 60 rpm as previously published (Drigny et al. 2014; Gayda et al. 2016). Gas exchanges were measured continuously at rest (during 3 min), during exercise, and recovery using a metabolic system (Oxycon Pro, Jaegger, Germany) as previously published. The calibration of the flow module was accomplished by introducing a calibrated volume of air at several flow rates with a 3‐liter pump. Gas analyzers were calibrated before each test using a standard certified commercial gas preparation (O_2_: 16%; CO_2_: 5%). Data were measured every four respiratory cycles during testing and then were averaged every 15 sec for minute ventilation ($\dot{V}$E, in L/min, BTPS), oxygen uptake ($\dot{V}O_{2}$, in L/min, STPD), carbon dioxide production ($\dot{V}$CO_2_, in L/min, STPD), end tidal pressure of CO_2_ (PETCO_2_: mmHg) (Drigny et al. 2014; Gayda et al. 2016). Maximal exercise test lasted until the attainment of one of the two primary maximal criteria: (A) a plateau of $\dot{V}O_{2}$ peak despite an increase in cadence, (B) R.E.R > 1.1 and one of the two secondary maximal criteria: (C) measured maximal heart rate attaining 95% of age‐predicted maximal heart rate, (D) inability to maintain the cycling cadence, (E) subject exhaustion with cessation caused by fatigue and/or other clinical symptoms (dyspnea, abnormal BP responses) or ECG abnormalities that required exercise cessation. The ventilatory threshold was determined using a combination of the V‐slope, ventilatory equivalents, and end‐tidal oxygen pressure methods (Gaskill et al. 2001). The highest $\dot{V}O_{2}$ peak value reached during the exercise phase of each test was considered as the $\dot{V}O_{2}$ peak and peak power output (PPO) was defined as the power output reached at the last fully completed stage (Drigny et al. 2014; Gayda et al. 2016).

#### Cardiac hemodynamics

Cardiac hemodynamics were measured continuously at rest (during 3 min), during exercise and recovery using a noninvasive impedance cardiography device (PhysioFlow^®^, Enduro model, Manatec, France), previously found to be valid, accurate, and reproducible at rest and during exercise in healthy subjects and coronary patients (Charloux et al. 2000; Richard et al. 2001; Vella et al. 2009, 2012; Drigny et al. 2014; Gayda et al. 2016). Data were averaged every 15 consecutive heartbeats for cardiac index (CI: in L/min/m^2^, stroke volume index (SVi: in mL/m^2^), heart rate (in beats/min), end diastolic and systolic volume index (EDVi and ESVi: in ml/m^2^), left cardiac work index (LCWi: in kg.m/m^2^) and systemic vascular resistance index (SVRi: in dynes/s/cm5/m^2^)(Gayda et al. 2012, 2016; Drigny et al. 2014).

#### Cerebral oxygenation/perfusion (COP)

The COP was measured using a near‐infrared spectroscopy (NIRS) system (Oxymon Mk III, Artinis Medical, Netherlands) during maximal exercise and recovery (Gremeaux et al. 2012; Drigny et al. 2014; Gayda et al. 2016). Optodes were placed on the left prefrontal cortical area between Fp1 and Fp3, according to the modified international EEG 10‐20 system (Gremeaux et al. 2012; Drigny et al. 2014; Gayda et al. 2016). Relative concentration changes (Δ*μ*mol/L) were measured from resting baseline of oxyhemoglobin (ΔO_2_Hb), deoxyhemoglobin (ΔHHb), total hemoglobin (ΔtHb = ΔO_2_Hb + ΔHHb) (Drigny et al. 2014; Gayda et al. 2016). The baseline period for exercise was set at the end of the 3‐min resting period, defined as 0 *μ*mol/L (Drigny et al. 2014; Gayda et al. 2016). For the recovery period, the reference point (0 *μ*mol/L) was set at the immediate start of the recovery period. Therefore, the cerebral re‐oxygenation/perfusion rate (ROPR) was defined by post‐exercise variation in the ΔO_2_Hb, ΔHHb and ΔtHb. Data were displayed in real time and stored on disk for off‐line analysis (Drigny et al. 2014; Gayda et al. 2016). Raw NIRS signals were filtered via the oxysoft/DAQ software (Artinis Medical, Netherlands) using a running average function with a filter width of 1(Drigny et al. 2014; Gayda et al. 2016). Thereafter, NIRS signals were exported into excel files with the oxysoft/DAQ software at 0.2 Hz for statistical treatment (Drigny et al. 2014; Gayda et al. 2016). During exercise test, optodes were secured with a tensor bandage wrapped around the forehead, a neoprene pad was place between the skin and the optodes plastic holder and ambient room light (dimmer) was reduced (Drigny et al. 2014; Gayda et al. 2016). To correct for scattering of photons in the tissue, a differential path‐length factor of 5.93 was used for the calculation of absolute concentration changes with an interoptode distance of 45 mm (Drigny et al. 2014; Gayda et al. 2016). Data were sampled at 10 Hz during the rest period (3 min), the exercise phase and the 5‐min recovery period (Drigny et al. 2014; Gayda et al. 2016).

### Statistical analysis

Results are presented as mean ± standard deviation except where otherwise indicated. Statistical analysis was performed using Statview software 5.0 (SAS, Cary) and GraphPad Prism 7.02 (GraphPad Software, Inc., La Jolla). Normal Gaussian distribution of the data was verified by the Shapiro–Wilk test. A one‐way ANOVA (groups) was used to compare maximal cardiopulmonary and hemodynamics parameters between: (1) AMHC and OB group and (2) AMHC vs. LF‐OB and HF‐OB groups. A two‐way ANOVA (groups x time) with repeated measure for time was performed to compare PETCO_2_, hemodynamics (CI, SBP, DBP) and cerebral NIRS parameters (ΔO_2_Hb, ΔHHb, ΔtHb) during exercise and recovery between: (1) AMHC and OB group and (2) AMHC versus LF‐OB and HF‐OB groups. A two‐way ANOVA (groups x time) with repeated measure for time was performed to compare PETCO_2_ and hemodynamics (CI, SBP, DBP) during exercise and recovery between obese subjects without medication and those taking medication. A Scheffé post hoc test was used to localize differences. Relationships between $\dot{V}O_{2}$ peak, ventilatory and cardiovascular parameters at peak effort were assessed with a Pearson coefficient of correlation (R). Statistical significance was set at *P* < 0.05 level for all analysis.

## Results

### Clinical and anthropometric characteristics

Table 1 describes the clinical and anthropometric characteristics of the AMHC, OB, LF‐OB and HF‐OB groups. As expected, the prevalence of cardiovascular risk factors was higher in the three obese groups (OB, LF‐OB, HF‐OB) versus AMHC for hypertension (51–55%), dyslipidemias (18–62%) and diabetes (22–59%). Regarding body composition, body mass, BMI, waist circumference and fat mass parameters were higher in the three obese groups (OB, LF‐OB, HF‐OB) versus AMHC (*P* < 0.001). Medication use was higher in the three obese groups (OB, LF‐OB, HF‐OB) particularly for statins, angiotensin receptor blockers and ACE inhibitors. Fasting glucose was higher in OB and LF‐OB versus AMHC (*P* < 0.01). HDL‐cholesterol and LDL‐cholesterol were lower and triglycerides higher in the three obese groups versus AMHC (*P* < 0.01).

**Table 1:** Baseline clinical characteristics of aged‐matched healthy controls (AMHC), obese subjects (OB), obese low‐fit (OB‐LF) and obese high‐fit (OB‐HF) groups

	AMHC (*n *=* *16)	OB (*n* = 54)	OB‐LF (*n *=* *27)	OB‐HF (*n *=* *27)
Age (years)	64 ± 4	62 ± 6	62 ± 7	61 ± 6
Height (cm)	172 ± 8	170 ± 9	169 ± 8	171 ± 10
Female sex	2 (33%)	16 (29%)	10 (37%)	6 (26%)
Smoking	0 (0%)	4 (7%)	2 (7%)	2 (7%)
Hypertension^1^	0 (0%)	29 (53%)	15 (55%)	14 (51%)
Diabetes^2^	0 (0%)	22 (40%)	16 (59%)	6 (22%)
History of dyslipidemia	0 (0%)	27 (50%)	17 (62%)	10 (18%)
Obesity^3^	0 (0%)	54 (100%)	27 (100%)	27 (100%)
Medication				
Beta–blockers	0 (0%)	8 (14%)	5 (18%)	3 (11%)
ACE inhibitors	0 (0%)	14 (25%)	7 (25%)	7 (25%)
Antiplatelet agents	0 (0%)	7 (12%)	5 (18%)	2 (7%)
Angiotensin receptor blockers	0 (0%)	16 (29%)	9 (33%)	7 (25%)
Statin	0 (0%)	24 (44%)	14 (51%)	10 (37%)
Calcium channel blockers	0 (0%)	6 (11%)	5 (18%)	1 (3%)
Hypoglycemic agents	0 (0%)	8 (14%)	8 (27%)	0 (0%)
Body composition				
Body mass (kg)	73 ± 8^a^^,^ §	93 ± 17	94 ± 17^b^^,^ §	91 ± 17^c^^,^ ‡
BMI (kg/m^2^)	24.5 ± 2.0^a^^,^ §	31.9 ± 5.0	32.9 ± 4.9^b^^,^ §	31.0 ± 5.0^c^^,^ §
Waist circumference (cm)	90 ± 7^a^^,^ §	110 ± 13	113 ± 13^b^^,^ §	107 ± 12^c^^,^ §
Lean body mass (kg)	57.8 ± 8.0	61.0 ± 11.9	60.1 ± 12.0	61.9 ± 11.9
FM percentage (%)	20.9 ± 4.2^a^^,^ §	34.1 ± 6.9	36.2 ± 6.2^b^^,^ §	32.2 ± 7.0^c^^,^ §
Total FM (kg)	15.2 ± 2.7^a^^,^ §	31.9 ± 9.6	34.3 ± 8.9^b^^,^ §	29.6 ± 9.9^c^^,^ §
Trunk FM percentage (%)	21.4 ± 3.7^a^^,^ §	34.9 ± 5.5	36.6 ± 4.9^b^^,^ §	33.2 ± 5.7^c^^,^ § ^,^ d ^,^ *
Trunk FM (kg)	8.9 ± 1.8^a^^,^ §	17.9 ± 5.6	19.1 ± 4.7^b^^,^ §	16.8 ± 6.3^c^^,^ §
Blood analysis				
Fasting glucose (mmol/L)	4.9 ± 0.3^a^^,^ †	6.1 ± 1.4	6.6 ± 1.6^b^^,^ §	5.7 ± 1.0
Total cholesterol (mmol/L)	4.92 ± 0.87	4.51 ± 1.61	4.33 ± 1.09	4.67 ± 1.96
HDL–cholesterol (mmol/L)	1.54 ± 0.42^a^^,^ †	1.24 ± 0.34	1.22 ± 0.38^b^^,^ †	1.25 ± 0.31^c^^,^ *
LDL–cholesterol (mmol/L)	3.13 ± 0.63^a^^,^ †	2.44 ± 0.77	2.44 ± 0.66^b^^,^ †	2.44 ± 0.90^c^^,^ †
Triglycerides (mmol/L)	0.73 ± 0.19^a^^,^ †	1.50 ± 0.90	1.58 ± 0.88^b^^,^ †	1.43 ± 0.93^c^^,^ †
Triglycerides/HDL	0.52 ± 0.25^a^^,^ †	1.35 ± 1.08	1.48 ± 1.1^b^^,^ †	1.25 ± 0.99

ACE, angiotensin–converting enzyme; CABG, coronary artery bypass grafting surgery; CHD, coronary heart disease; MI, myocardial infarction; PCI, percutaneous coronary intervention; BMI, body mass index; FM, fat mass; SBP, systolic blood pressure; DBP, diastolic blood pressure.

Group effect: ^a^AMHC versus OB, ^b^AMHC versus OB‐LF, ^c^AMHC versus OB‐HF, ^d^OB‐LF versus OB‐HF.

^1^Rest SBP > 130 mmHg; ^2^glucose > 7 mmol/l; ^3^Body fat mass %: women > 35% and men > 25%.

*P < 0.05, ^†^ *P* < 0.01, ^‡^ *P* < 0.001, ^§^ *P* < 0.0001.

### Cardiopulmonary exercise testing parameters

The cardiopulmonary exercise testing parameters measured at rest, ventilatory threshold (VT) and peak effort are described in details in Table 2 **.** Resting HR and SBP were higher in the three obese groups versus AMHC (*P* < 0.05). $\dot{V}O_{2}$ uptake and power at VT were higher in AMHC versus OB and LF‐OB (*P* < 0.001). $\dot{V}O_{2}$ peak, % of $\dot{V}O_{2}$ peak predicted and peak power were higher in AMHC versus the OB and LF‐OB (*P* < 0.05). $\dot{V}E$ peak, % of $\dot{V}E$ peak predicted and tidal volume (TV) were lower in LF‐OB versus AMHC and HF‐OB (*P* < 0.05). Breathing frequency (Bf) was higher in HF‐OB versus LF‐OB (*P* < 0.05). At peak effort, cardiac output (CO), cardiac index (CI), stroke volume index (SVi), end diastolic and systolic volume (EDVi, ESVi) and systemic vascular resistance index (SVRi) did not differ between groups. Peak HR was lower in LF‐OB versus AMHC and HF‐OB (*P* < 0.05) and heart rate recovery (ΔHRR at 1 min) was higher in HF‐OB versus LF‐OB (*P* < 0.05). Relationship between $\dot{V}O_{2}$ peak, ventilatory and cardiovascular parameters (measured at peak effort) are described in Table 3. In AMHC, $\dot{V}O_{2}$ peak was significantly correlated with ventilation ($\dot{V}E$), breathing frequency (Bf), cardiac output (CO), cardiac index (CI), end diastolic volume index (EDVi), end systolic volume index (ESVi) and left cardiac work index (LCWi). In obese subjects (OB), $\dot{V}O_{2}$ peak was significantly correlated with ventilation ($\dot{V}E$), tidal volume (TV), breathing frequency (Bf), cardiac index (CI) and left cardiac work index (LCWi).

**Table 2:** Cardiopulmonary and hemodynamic variables at rest and during exercise testing in aged‐matched healthy controls (AMHC), obese subjects (OB), obese low‐fit (OB‐LF) and obese high‐fit (OB‐HF) groups

Cardiopulmonary and hemodynamic variables	AMHC (*n *=* *16)	OB (*n *=* *54)	OB‐LF (*n *=* *27)	OB‐HF (*n *=* *27)	ANOVA *P* value	
					AMHC vs. OB	AMHC vs. OB‐LF vs. OB‐HF
Rest						
Resting heart rate	63 ± 7^a^^,^ ‡	77 ± 14	76 ± 15^b^^,^ †	77 ± 13^c^^,^ †	0.0006	0.0031
Rest SBP (mmHg)	119 ± 15^a^^,^ †	130 ± 11	130 ± 13^b^^,^ *	129 ± 9^c^^,^ *	0.0023	0.0089
Rest DBP (mmHg)	73 ± 10	75 ± 12	74 ± 16	75 ± 8	0.6203	0.8312
At ventilatory threshold						
$\dot{V}$O_2_ uptake (ml/min/LBM)	33.3 ± 5.2^a^^,^ ‡	27.4 ± 5.7	22.9 ± 3.6^b^^,^ §	31.4 ± 4.0^d^^,^ §	<0.0001	0.0009
Power (Watts)	148 ± 43^a^^,^ ‡	110 ± 43	83 ± 28^b^^,^ §	135 ± 39^d^^,^ §	0.0050	<0.0001
At peak						
$\dot{V}$O_2_ peak (ml/min/LBM)	44.5 ± 6.0^a^^,^ †	37.3 ± 7.7	30.9 ± 4.4^b^^,^ §	43.6 ± 4.3^d^^,^ §	0.0011	<0.0001
% of $\dot{V}$O_2_ peak predicted	139 ± 16^a^^,^ †	110 ± 35	98 ± 21^b^^,^ ‡	122 ± 42^d^^,^ *	0.0049	<0.0001
$\dot{V}$CO_2_ (ml/min)	2918 ± 719	2613 ± 817	2091 ± 593^b^^,^ †	3096 ± 693^d^^,^ §	0.2079	<0.0001
R.E.R	1.15 ± 0.06	1.14 ± 0.07	1.15 ± 0.07	1.13 ± 0.08	0.6064	0.4706
Peak power (Watts)	204 ± 47^a^^,^ *	162 ± 60	122 ± 35^b^^,^ §	198 ± 55^d^^,^ §	0.0131	<0.0001
$\dot{V}$E peak (l/min)	94 ± 30	82 ± 26	66 ± 20^b^^,^ †	96 ± 23^d^^,^ ‡	0.1406	<0.0001
% of $\dot{V}$E peak predicted	139 ± 35	126 ± 36	105 ± 29^b^^,^ †	145 ± 31^d^^,^ §	0.2263	<0.0001
$\dot{V}$E/$\dot{V}$CO_2_	32 ± 6	31 ± 4	31 ± 4	32 ± 5	0.6754	0.8180
TV (liters)	2.58 ± 0.45	2.25 ± 0.58	2.04 ± 0.51^b^^,^ *	2.45 ± 0.58^d^^,^ *	0.0549	0.0040
Bf (resp/min)	37 ± 11	36 ± 8	33 ± 8	40 ± 8^d^^,^ *	0.9798	0.0272
CO (L/min)	15.5 ± 3.4	15.0 ± 3.4	14.0 ± 3.6	15.9 ± 2.9	0.5871	0.1082
CI max (L/min/m^2^)	7.9 ± 1.7	7.3 ± 1.7	6.8 ± 1.8	7.8 ± 1.6	0.2220	0.0513
C(a‐v)O_2_ (mL/100 mL)	15.1 ± 3.1	16.0 ± 3.5	15.9 ± 3.1	16.1 ± 4.0	0.6216	0.6618
SVi (mL/m^2^)	54 ± 6	53 ± 11	51 ± 14	55 ± 8	0.6524	0.2433
EDVi (mL/m^2^)	119 ± 31	129 ± 71	121 ± 33	136 ± 96	0.6216	0.6296
ESVi (mL/m^2^)	64 ± 32	66 ± 34	67 ± 35	66 ± 33	0.8378	0.9591
LCWi (kg.m/m²)	11.8 ± 3.1	11.4 ± 3.2	10.6 ± 3.1	12.2 ± 3.1	0.6842	0.1422
SVRi (dyn.s/cm^5^.m²)	616 ± 156	666 ± 198	719 ± 250	615 ± 113	0.3568	0.0819
Peak HR (bpm)	154 ± 10	147 ± 24	137 ± 25^b^^,^ *	158 ± 18^d^^,^ †	0.2819	0.0008
ΔHRR at 1 min (bpm)	−20 ± 6	−24 ± 14	−20 ± 12	−27 ± 14^d^^,^ *	0.3363	0.0482
Max SBP (mmHg)	186 ± 24	193 ± 27	196 ± 29	191 ± 26	0.3359	0.5050
Max DBP (mmHg)	81 ± 11	79 ± 14	76 ± 18	81 ± 7	0.5575	0.3585

SBP, systolic blood pressure; DBP, diastolic blood pressure; LBM, lean body mass; R.E.R, respiratory exchange ratio; TV, tidal volume; Bf, breathing frequency; CI, cardiac index; C(a‐v)O_2,_ arterio venous difference; SVi, stroke volume index; LCWi, left cardiac work index; SVRi, systemic vascular resistance index; HR, heart rate; HRR, heart rate recovery.

Group effect: ^a^AMHC versus OB, ^b^AMHC versus OB‐LF, ^c^AMHC versus OB‐HF, ^d^OB‐LF versus OB‐HF.

*P < 0.05, ^†^ *P* < 0.01, ^‡^ *P* < 0.001, ^§^ *P* < 0.0001.

**Table 3:** Correlation between $\dot{V}O_{2}$ peak, pulmonary and cardiovascular parameters (at peak) in aged‐matched healthy controls (AMHC) and obese subjects (OB)**.**

	$\dot{V}O_{2}$ peak (ml/min/LBM)	$\dot{V}O_{2}$ peak (ml/min/LBM)
At peak	AMHC	OB
$\dot{V}$E	*R* = 0.66, *P* = 0.0085	*R* = 0.64, *P* < 0.0001
VT	*R* = 0.17, *P* = 0.5676	*R* = 0.43, *P* = 0.0011
Bf	*R* = 0.56, *P* = 0.0351	*R* = 0.39, *P* = 0.0035
CO	*R* = 0.69, *P* = 0.0019	*R* = 0.25, *P* = 0.0595
CI	*R* = 0.67, *P* = 0.0031	*R* = 0.29, *P* = 0.0312
C(a‐v)O_2_	*R* = −0.24, *P* = 0.3744	*R* = 0.09, *P* = 0.4986
SVi	*R* = 0.36, *P* = 0.1640	*R* = 0.03, *P* = 0.7880
EDVi	*R* = −0.53, *P* = 0.0308	*R* = −0.02, *P* = 0.8871
ESVi	*R* = −0.60, *P* = 0.0112	*R* = −0.03, *P* = 0.8160
LCWi	*R* = 0.64, *P* = 0.0060	*R* = 0.28, *P* = 0.0338
SBP	*R* = 0.08, *P* = 0.7811	*R* = 0.01, *P* = 0.9099
DBP	*R* = 0.14, *P* = 0.6346	*R* = 0.14, *P* = 0.2989

$\dot{V}$Emax, maximal ventilation; TV, tidal volume; Bf, breathing frequency; CO, cardiac ouput; CI, cardiac index; C(a‐v)O_2_, arterio venous difference; SVi, stroke volume index; EDVi, end diastolic volume index; ESVi, end systolic volume index; LCWi, left cardiac work index; SBP, systolic blood pressure; DBP, diastolic blood pressure.

### End tidal pressure of CO_2_, cardiac index and blood pressure during exercise and recovery

**Figure 1:**
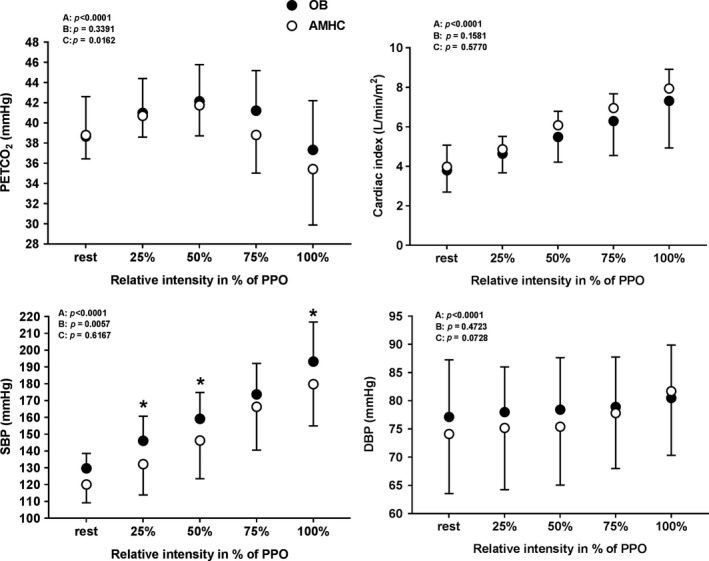
PETCO_2_, cardiac index, systolic and diastolic blood pressure during exercise in aged‐matched healthy controls (AMHC) and obese subjects (OB). ANOVA effect: A=group, B=time, C=interaction; Post hoc group effect: *= *P* < 0.05.

**Figure 2:**
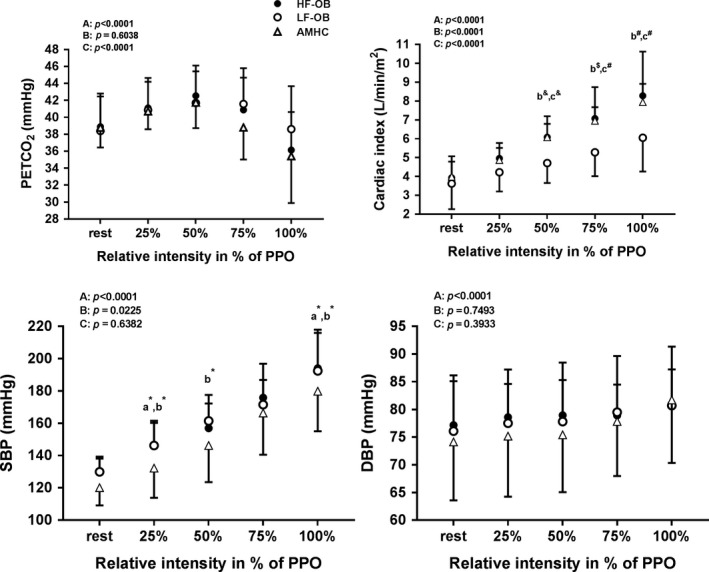
PETCO_2_, cardiac index, systolic and diastolic blood pressure during exercise in aged‐matched healthy controls (AMHC), low‐fit obese group (LF‐OB) and high‐fit obese group (HF‐OB). ANOVA effect: A=group, B=time, C=interaction; Post hoc group effect: a=AMHC versus LF‐OB, b=LF‐OB versus HF‐OB, c=AMHC versus HF‐OB, *= *P* < 0.05, &=*P* < 0.01, $=*P* < 0.001, #=*P* < 0.0001.

**Figure 3:**
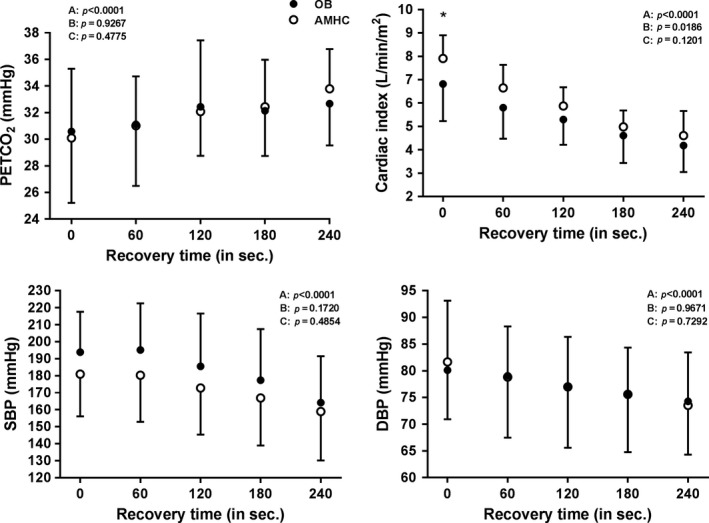
PETCO_2_, cardiac index, systolic and diastolic blood pressure during recovery in aged‐matched healthy controls (AMHC) and obese subjects (OB). ANOVA effect: A=group, B=time, C=interaction; Post hoc group effect: *=*P* < 0.05.

**Figure 4:**
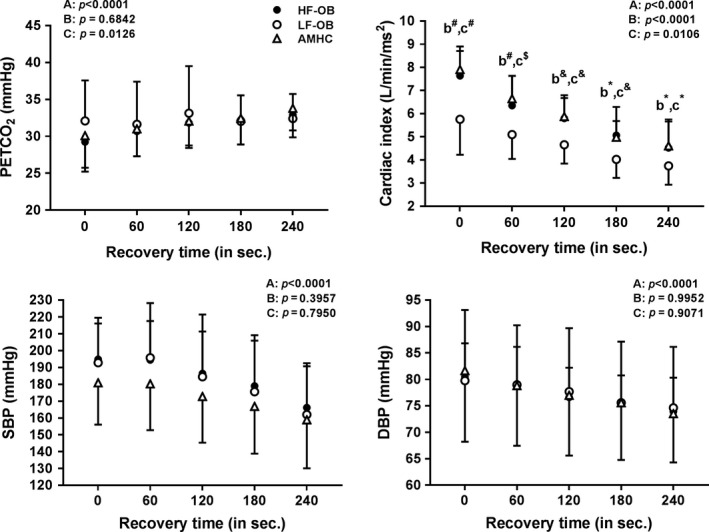
PETCO_2_, cardiac index, systolic and diastolic blood pressure during exercise in aged‐matched healthy controls (AMHC), low‐fit obese group (LF‐OB) and high‐fit obese group (HF‐OB). ANOVA effect: A=group, B=time, C=interaction; Post hoc group effect: b=LF‐OB vs. HF‐OB, c=AMHC vs HF‐OB, *=*P* < 0.05, &=*P* < 0.01, $=*P* < 0.001, #=*P* < 0.0001.

Figures 1, 2, 3, 4 describe end tidal pressure of CO_2_, cardiac index, systolic and diastolic blood pressure during exercise and recovery. During exercise, PETCO_2_ did not differ among OB, LF‐OB and HF‐OB groups versus AMHC **(**Fig. 1 and 2 **)**. During exercise, cardiac index did not differ among OB and AMHC **(**Fig.**** 1 **)** but was higher in AMHC and HF‐OB versus LF‐OB at 50%, 75% and 100% of PPO (*P* < 0.05, Fig.**** 2). During exercise, SBP was higher at 25%, 50%, and 100% of PPO in OB and in LF‐OB versus AMHC (*P* < 0.05) and higher at 25% and 100% of PPO in HF‐OB versus AMHC (Figs. 1 and 2 **)**. During recovery, PECTCO_2_, SBP and DBP did not differ among the groups (OB, LF‐OB, HF‐OB and AMHC) **(**Figs. 3 and 4 **)**. During recovery, cardiac index was lower in OB (at 0 sec) and in LF‐OB (from 0 to 120 sec) as compared to AMHC and HF‐OB (*P* < 0.05) **(**Figs.**** 3 and 4 **).** During exercise and recovery, PETCO_2_, SBP and DBP (*P* > 0.05) did not differ between obese subjects taking medication versus those without medication. During exercise and recovery, cardiac index was lower (*P* < 0.01) in obese subjects taking medication versus those without medication.

### Left prefrontal NIRS parameters during exercise and recovery

**Figure 5:**
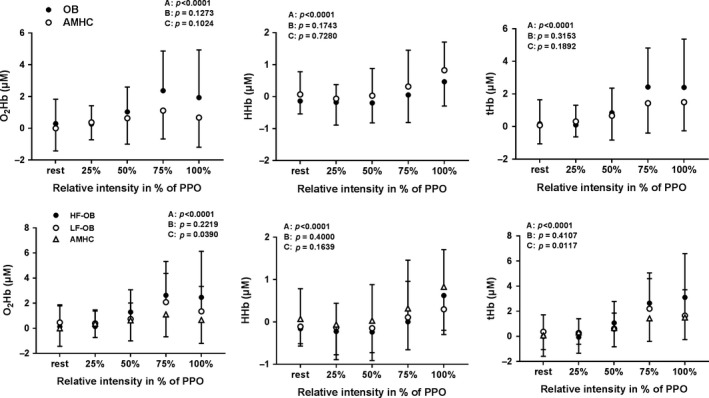
Cerebral oxygenation/perfusion during exercise in age‐matched healthy controls (AMHC), obese subjects (OB), low‐fit obese group (LF‐OB) and high‐fit obese group (HF‐OB). ANOVA effect: A=group, B=time, C=interaction.

**Figure 6:**
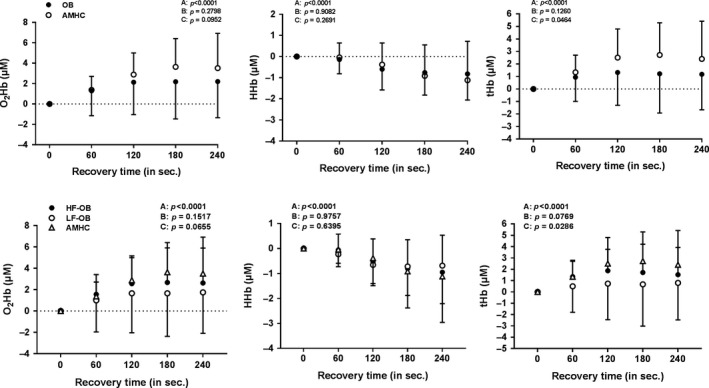
Cerebral oxygenation/perfusion during recovery in age‐matched healthy controls (AMHC), obese subjects (OB), low‐fit obese group (LF‐OB) and high‐fit obese group (HF‐OB). ANOVA effect: A=group, B=time, C=interaction.

Figure 5 describes left prefrontal NIRS parameters during exercise in AMHC, OB, LF‐OB and HF‐OB. During exercise, no differences were found for ΔO_2_Hb, ΔHHb and ΔtHb between AMHC and OB, LF‐OB and HF‐OB (Fig. 5). Figure 6 describes left prefrontal NIRS parameters during recovery in AMHC, OB, LF‐OB and HF‐OB. During recovery, no differences were found for ΔO_2_Hb, ΔHHb and ΔtHb between AMHC and OB, LF‐OB and HF‐OB (Fig. 6).

## Discussion

### Major findings

The main findings were: (1) Compared to AMHC, obese subjects (OB) have a lower $\dot{V}O_{2}$ peak, higher systolic blood pressure, but similar cardiac output, cerebral vasoreactivity by CO_2_ (PETCO_2_) and cerebral oxygenation/perfusion during incremental maximal exercise. (2) During recovery, obese subjects (OB) had lower cardiac index (only at 0 min) but a similar PETCO_2_, blood pressure and cerebral re‐oxygenation/perfusion rate (ΔO_2_Hb, ΔtHb) compared to AMHC. (3) During exercise, the LF‐OB group showed a lower cardiac output, higher SBP but similar cerebral vasoreactivity by CO_2_ (PETCO_2_) and cerebral oxygenation/perfusion compared to the other two groups (AMHC, HF‐OB). (4) During exercise, the HF‐OB group had higher SBP but similar cardiac output, cerebral vasoreactivity by CO_2_ (PETCO_2_) and cerebral oxygenation/perfusion compared to the other two groups (AMHC, HF‐OB). (5) During recovery, the LF‐OB and HF‐OB had similar cerebral vasoreactivity by CO_2_ (PETCO_2_), blood pressure and cerebral re‐oxygenation/perfusion rate (ΔO_2_Hb, ΔtHb) compared to AMHC. Only cardiac output was lower in the LF‐OB during recovery versus the other two groups (AMHC, HF‐OB).

Although there were group differences in certain cardiovascular measures, cerebral hemodynamics during exercise and recovery were not different between obese groups (OB, LF‐OB, HF‐OB) and AMHC. To the best of our knowledge, this study is the first to integrate a simultaneous measure of cerebral hemodynamics and their determinants (PETCO_2_, cardiac output and blood pressure) during incremental maximal exercise and its recovery in older obese subjects with different level of aerobic fitness. This study underlies the importance of aerobic fitness in obese subjects and its protective effects on cardiovascular function during exercise and recovery.

### Cardiopulmonary and hemodynamic variables during exercise

Compared to AMHC, obese subjects (OB) and low‐fit obese (LF‐OB) showed a lower maximal aerobic power ($\dot{V}O_{2}$ peak) and peak power output. As well, the LF‐OB had lower peak ventilatory function versus AMHC. The lower $\dot{V}O_{2}$ peak (normalized by LBM) found in OB and LF‐OB is in agreement with one previous study (Fournier et al. 2014) but is inconsistent with results from two other studies (Vella et al. 2011, 2012). The C(a‐v)O_2_ between our groups (OB, LF‐OB, HF‐OB and AMHC) was not different which is in disagreement with previous studies (Vella et al. 2011, 2012; Fournier et al. 2014). An impaired muscle oxygen extraction observed in previous studies was proposed to be due to a lower mitochondrial density/function, oxygen transport capacity and to endothelial dysfunction (Vella et al. 2011, 2012). The lower $\dot{V}O_{2}$ peak particularly in LF‐OB could be explained by a combined reduced pulmonary function ($\dot{V}$E and TV) and maximal cardiac index compared to AMHC (Table 2 and Fig. 1 to 4). Therefore, high aerobic fitness seemed to be a protective factor regarding impairment of pulmonary and cardiac function in the HF‐OB group.

Compared to AMHC, the three obese groups presented a similar maximal indexed cardiac output and stroke volume at peak effort, with a trend (*P* = 0.051) for lower CImax in LF‐OB (Table 2). However, low‐fit obese (LF‐OB) showed a reduced cardiac index during incremental exercise (50–100% of PPO) as compared to AHMC and HF‐OB (Fig.**** 2). Similar indexed end diastolic and systolic volumes (ESVi and EDVi) and systemic vascular resistance were shown among obese groups at peak exercise versus AMHC. Conflicting results have been reported on cardiac index during exercise in obese subjects. One study (Vella et al. 2012) reported higher cardiac index (normalized by resting HR and body surface area) during submaximal exercise and similar maximal cardiac index between young obese and nonobese controls. A second study reported lower cardiac index during submaximal and maximal exercise in OB versus AMHC (Fournier et al. 2014). As well, previous studies on ESVi and EDVi at peak effort in obese subjects are conflicting (Vella et al. 2012; Fournier et al. 2014). Founier et al. showed a higher ESVi but similar EDVi in obese subjects versus AMHC at peak effort. In contrast, Vella et al. (2012) showed no difference in EDVi at peak effort in young obese subjects versus AMHC. Those discrepancies might be explained by different factors including the age of the obese subjects (younger vs. older), their aerobic fitness level ($\dot{V}O_{2}$ peak) and the hemodynamics measurement method (echocardiography vs. impedance cardiography). Previous studies suggest that resting diastolic dysfunction is present in early stages of obesity, due to an increased blood volume and cardiac preload at rest, responsible of a gradual enlargement and thickening of the left ventricle (Schuster et al. 2012; Vella et al. 2012; Fournier et al. 2014). The similar cardiac index in the HF‐OB during incremental exercise (Fig.**** 2) as compared to AMHC suggests that a high aerobic fitness ($\dot{V}O_{2}$ peak) in obese subjects may normalize and preserve cardiac function (Schuster et al. 2012). This protective effect of fitness on cardiac function in obese subjects is clinically relevant, and may protect them from cardiovascular diseases such as heart failure for example. In our subanalysis, this positive effect was reinforced by the fact that obese subjects taking medication had lower cardiac function during exercise and recovery versus the obese subjects not taking medication. This may reflect that obese subjects taking medication may have a higher cardiovascular risk profile (Table 1) and therefore a lower cardiac function as compared to obese subjects not taking medication. Previous studies indicate that aerobic exercise training reduces resting end systolic volumes (Schuster et al. 2012) and improves systolic and/or diastolic function at rest (Ingul et al. 2010; Schuster et al. 2012; Hollekim‐Strand et al. 2014), less is known about the effects of those intervention on cardiac function during exercise.

### Cardiac and cerebral hemodynamics during and after exercise

During exercise, cerebral oxygenation/perfusion was similar between the obese groups (OB, LF‐OB, HF‐OB) and AMHC for all intensities. Our results are in agreement with a previous study showing similar COP (measured by NIRS) during submaximal cycling exercise between young obese adults and nonobese controls (Cavuoto and Maikala 2016). Previous findings in healthy young adults demonstrated a higher a COP in the fittest subjects (Brugniaux et al. 2014; Oussaidene et al. 2015), but this effect was not found in our HF‐OB group. Another study showed a reduced cerebral oxygenation (measured by NIRS) in young obese adults during repetitive incremental lifting to exhaustion as compared to nonobese controls (Cavuoto and Maikala 2015). Therefore, aging (Fisher et al. 2013), lower aerobic fitness (Rooks et al. 2010; Brugniaux et al. 2014; Oussaidene et al. 2015) and cardiac disease (Koike et al. 2004a, 2004b; Fu et al. 2013) were shown to reduce COP during exercise. To explain similar COP in obese groups during exercise, variables implicated in cerebral hemodynamics regulation during exercise (PETCO_2_, cardiac output and blood pressure) were analyzed (Ogoh and Ainslie 2009). First, it is to note that CO_2_ (reflected by PETCO_2_) is a powerful vasodilator of cerebral vessels during exercise and that this variable was not different among the obese groups and healthy controls (Fig.**** 1, 2). Secondly, systolic blood pressure during exercise was higher in obese groups (Fig.**** 1, 2) and therefore would have helped to maintain perfusion particularly in LF‐OB despite a lower cardiac index during exercise (Fig.**** 1, 2). In addition, we may speculate that other factors, such as hematological ones and inter‐individual variability in NIRS could have influenced our results in our obese subjects. For example, higher blood volume and hemoglobin concentration have been previously reported in obese subjects (Vella et al. 2012; Kishi et al. 2014), and could be responsible for a similar COP reported during exercise in the obese groups but this requires further experimentation.

During recovery, cerebral re‐oxygenation/perfusion rate (ROPR) was also similar in the obese groups (OB, LF‐OB and HF‐OB) as compared to AMHC. Among variables implicated in cerebral hemodynamics regulation (PETCO_2_, cardiac output, blood pressure), only cardiac index was reduced in OB (1st min) and LF‐OB (0–240 sec) during recovery. This indicates that obesity does not seem to have negative effects on cerebral hemodynamics and auto‐regulation after exercise for the prefrontal area measured. As well, our results underlie that post exercise cardiac functions seemed preserved in HF‐OB group versus AMHC. Similarly, hematological factors and inter‐individual variability in NIRS could have also influenced our results in our obese subjects during recovery. Our results agree with previously studies reporting similar cerebral hemodynamics after exercise in obese subjects versus nonobese controls (Cavuoto and Maikala 2015, 2016). Other studies have reported a reduced cerebral hemodynamics during recovery in cardiac patients (Koike et al. 2004b; Gayda et al. 2016). During recovery, cerebral arterial vasodilation must be triggered to re‐increased oxygenation and perfusion (Brown et al. 2010; Davenport et al. 2012), nitric oxide (NO) being an important mediator of cerebral endothelium‐dependent vasodilation.

### Limitations

Our study has several limitations, including the enrolment of healthy and obese subjects recruited in a single center, hence inducing a potential recruitment bias. A majority of the participants were mainly male, and frequenting our cardiovascular prevention centre. Cerebral hemodynamics were assessed using NIRS at the left prefrontal area implicating a very limited spatial resolution and a relatively superficial brain tissue measurement (light penetration ≈ 2.25 cm)(Gayda et al. 2016). Therefore, our results may differ from other more invasive and global measurement of cerebral hemodynamics (ex: catheters, transcranial Doppler or NIRS with more extensive brain coverage) or from other brain regions. Finally, cerebral NIRS signals can be influenced by extracranial tissue skin blood flow during exercise (Miyazawa et al. 2013). To limit this influence, the room temperature was standardized (thermo neutral environment: 20°C) and we have used a more important interoptode distance (4.5 cm) to allow deeper light penetration into intracranial tissues (Germon et al. 1999; Gayda et al. 2016).

## Conclusion

The main findings of this study were: (1) Obese subjects groups (OB, LF‐OB, HF‐OB) have a similar cerebral vasoreactivity by CO_2_ (PETCO_2_) and cerebral hemodynamics (COP) but higher systolic blood pressure during exercise versus AMHC. (2) During recovery, obese subjects groups (OB, LF‐OB, HF‐OB) have a similar cerebral hemodynamics (ROPR) versus AMHC. (3) During exercise and recovery, low‐fit obese (LF‐OB) have a lower cardiac function as compared to AMHC. (4) During exercise and recovery, high‐fit obese (HF‐OB) have a similar cardiac function versus AMHC. Our study suggests that a higher aerobic fitness in obese subjects might have a protective effect on cardiac and pulmonary function during exercise and recovery. In perspective, future studies with other methods of cardiac and cerebral hemodynamics measurement (such as: echocardiography and transcranial Doppler) would be required to confirm our results in obese populations with different levels of fitness and/or cardiovascular risk factors.

## Conflict of Interest

None declared.
